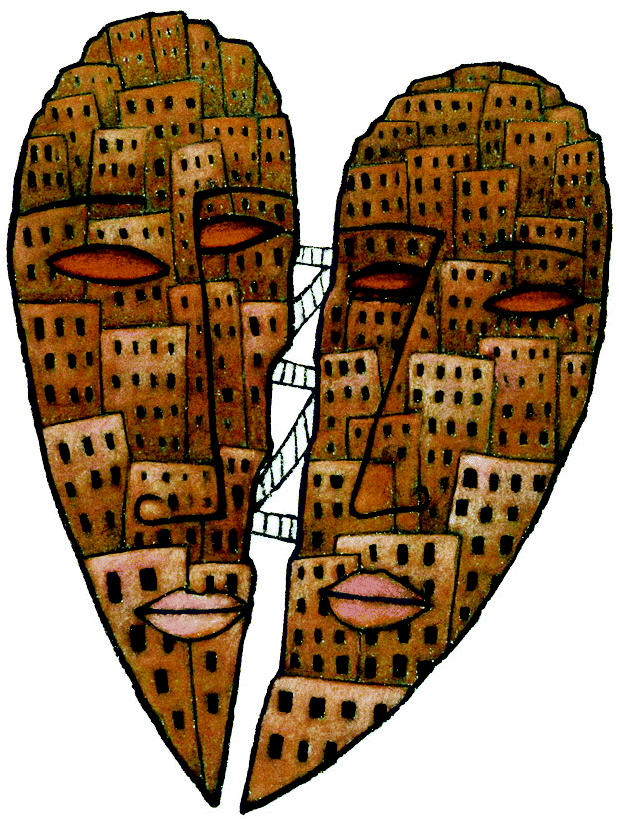# Environmental Justice: Young Hearts Suffer in Poorer Countries

**Published:** 2004-11

**Authors:** Carol Potera

Cardiovascular disease (CVD) is a well-known killer of older people in affluent countries. In developing countries, however, the disease is striking a younger age group. In India, South Africa, Brazil, and the Russian republic of Tatarstan, people aged 35–65 die from CVD significantly more often than counterparts in the United States, according to a report released recently by Columbia University’s Earth Institute.

CVD is on the rise in developing nations for the same reasons that made it a killer in the west: a rise in cigarette smoking, a higher-fat diet, and lack of physical exercise. The CVD death rate in developing areas is reminiscent of that experienced in the United States in the 1950s and 1960s before effective public health measures, like warnings about the dangers of smoking and treatment for hypertension, became common. But such measures “have not yet occurred in developing countries, and treatment is often unavailable,” says epidemiologist Stephen Leeder of the University of Sydney in Australia, who coordinated the project while a visiting fellow at Columbia University.

Leeder’s team combined available death rate and workforce data from five representative middle- and low-income regions to estimate the economic impact of CVD on society. Their report, released in April 2004, reveals a silent epidemic affecting both women and men of working age.

In Tatarstan, CVD deaths among men aged 35–64 have soared 70% in just 20 years. Among women aged 15–34, four times more die from CVD than from pregnancy-related problems—a surprise, given that CVD is rarely considered a woman’s disease in developing nations. China’s CVD death rate currently mirrors that of the United States but is expected to be twice the U.S. rate by 2030, when half of the 9 million projected Chinese CVD deaths will be among people aged 35–64. In Brazilians aged 35–44, the male death rate from CVD is 30% higher and the female death rate 75% higher than for the same age group in the United States. In South Africa, CVD ranks third as cause of death in women and sixth for men.

The report’s title, *A Race Against Time*, refers to a 20-year window of opportunity to tackle the problem. “Younger people can be educated about lifestyle changes and treated with drugs,” says Leeder. If action is not taken now, the health costs in 20 years as these people reach end-stage disease “will be stupendous,” he warns.

As world health organizations struggle to finance treatments for infectious diseases such as malaria and AIDS, the report reminds us that “we need to pay attention to chronic diseases like heart disease,” says Daniel Fox, president of the Milbank Memorial Fund, which works with decision makers to bring the best available evidence to bear on health care and public health policy. The report is “analytically tight,” says Fox, and suggests that the economic and social impact of heart disease in the next generation may dwarf that of communicable diseases.

## Figures and Tables

**Figure:**